# Supplementary material for: Unveiling the Bioactive Potential of the Invasive Jellyfish Phyllorhiza punctata Through Integrative Transcriptomic and Proteomic Analyses
Source: Biomolecules. 2025 Aug 4;15(8):1121. doi: 10.3390/biom15081121 (PMC12383608; doi:10.3390/biom15081121)
Supplement: Supplementary file 1 [file biomolecules-15-01121-s001.zip › Suplementary Material/FileS3/PEPFOLD4/XP_065063258_5622/clusters.html]

 PEP-FOLD clusters


Download archive of all models  here   

|  |  |  |
| --- | --- | --- |
| # | file | sOPEP |
| # cluster 1 - Size: 1 |  |  |
|  | XP\_065063258\_5622-model1 | -7.63684 |
| # cluster 2 - Size: 1 |  |  |
|  | XP\_065063258\_5622-model2 | -7.40604 |
| # cluster 3 - Size: 1 |  |  |
|  | XP\_065063258\_5622-model3 | -7.14083 |
| # cluster 4 - Size: 2 |  |  |
|  | XP\_065063258\_5622-model4 | -7.03454 |
|  | XP\_065063258\_5622-model4.1 | -6.88565 |
| # cluster 5 - Size: 1 |  |  |
|  | XP\_065063258\_5622-model5 | -6.82177 |
| # cluster 6 - Size: 1 |  |  |
|  | XP\_065063258\_5622-model6 | -6.71088 |
| # cluster 7 - Size: 1 |  |  |
|  | XP\_065063258\_5622-model7 | -4.84301 |
| # cluster 8 - Size: 1 |  |  |
|  | XP\_065063258\_5622-model8 | -4.73399 |
| # cluster 9 - Size: 3 |  |  |
|  | XP\_065063258\_5622-model9 | -4.55444 |
|  | XP\_065063258\_5622-model9.1 | -4.22039 |
|  | XP\_065063258\_5622-model9.2 | -4.02516 |
| # cluster 10 - Size: 3 |  |  |
|  | XP\_065063258\_5622-model10 | -4.52634 |
|  | XP\_065063258\_5622-model10.1 | -3.79497 |
|  | XP\_065063258\_5622-model10.2 | -3.18033 |
| # cluster 11 - Size: 3 |  |  |
|  | XP\_065063258\_5622-model11 | -4.31075 |
|  | XP\_065063258\_5622-model11.1 | -4.12749 |
|  | XP\_065063258\_5622-model11.2 | -3.86361 |
| # cluster 12 - Size: 4 |  |  |
|  | XP\_065063258\_5622-model12 | -4.2809 |
|  | XP\_065063258\_5622-model12.1 | -3.9686 |
|  | XP\_065063258\_5622-model12.2 | -3.69612 |
|  | XP\_065063258\_5622-model12.3 | -3.52747 |
| # cluster 13 - Size: 3 |  |  |
|  | XP\_065063258\_5622-model13 | -4.20448 |
|  | XP\_065063258\_5622-model13.1 | -3.72736 |
|  | XP\_065063258\_5622-model13.2 | -3.61519 |
| # cluster 14 - Size: 1 |  |  |
|  | XP\_065063258\_5622-model14 | -4.16327 |
| # cluster 15 - Size: 2 |  |  |
|  | XP\_065063258\_5622-model15 | -4.12864 |
|  | XP\_065063258\_5622-model15.1 | -3.52257 |
| # cluster 16 - Size: 3 |  |  |
|  | XP\_065063258\_5622-model16 | -4.10442 |
|  | XP\_065063258\_5622-model16.1 | -3.75709 |
|  | XP\_065063258\_5622-model16.2 | -3.46639 |
| # cluster 17 - Size: 1 |  |  |
|  | XP\_065063258\_5622-model17 | -4.09533 |
| # cluster 18 - Size: 1 |  |  |
|  | XP\_065063258\_5622-model18 | -4.09519 |
| # cluster 19 - Size: 1 |  |  |
|  | XP\_065063258\_5622-model19 | -4.0569 |
| # cluster 20 - Size: 1 |  |  |
|  | XP\_065063258\_5622-model20 | -4.03458 |
| # cluster 21 - Size: 4 |  |  |
|  | XP\_065063258\_5622-model21 | -4.02579 |
|  | XP\_065063258\_5622-model21.1 | -3.97994 |
|  | XP\_065063258\_5622-model21.2 | -3.82147 |
|  | XP\_065063258\_5622-model21.3 | -3.59155 |
| # cluster 22 - Size: 1 |  |  |
|  | XP\_065063258\_5622-model22 | -3.99987 |
| # cluster 23 - Size: 4 |  |  |
|  | XP\_065063258\_5622-model23 | -3.99526 |
|  | XP\_065063258\_5622-model23.1 | -3.79146 |
|  | XP\_065063258\_5622-model23.2 | -3.74532 |
|  | XP\_065063258\_5622-model23.3 | -3.36173 |
| # cluster 24 - Size: 1 |  |  |
|  | XP\_065063258\_5622-model24 | -3.95751 |
| # cluster 25 - Size: 4 |  |  |
|  | XP\_065063258\_5622-model25 | -3.95744 |
|  | XP\_065063258\_5622-model25.1 | -3.92229 |
|  | XP\_065063258\_5622-model25.2 | -3.7375 |
|  | XP\_065063258\_5622-model25.3 | -3.58323 |
| # cluster 26 - Size: 1 |  |  |
|  | XP\_065063258\_5622-model26 | -3.95129 |
| # cluster 27 - Size: 1 |  |  |
|  | XP\_065063258\_5622-model27 | -3.92895 |
| # cluster 28 - Size: 1 |  |  |
|  | XP\_065063258\_5622-model28 | -3.92634 |
| # cluster 29 - Size: 3 |  |  |
|  | XP\_065063258\_5622-model29 | -3.92204 |
|  | XP\_065063258\_5622-model29.1 | -3.67182 |
|  | XP\_065063258\_5622-model29.2 | -3.52884 |
| # cluster 30 - Size: 3 |  |  |
|  | XP\_065063258\_5622-model30 | -3.89627 |
|  | XP\_065063258\_5622-model30.1 | -3.41609 |
|  | XP\_065063258\_5622-model30.2 | -3.32439 |
| # cluster 31 - Size: 4 |  |  |
|  | XP\_065063258\_5622-model31 | -3.85351 |
|  | XP\_065063258\_5622-model31.1 | -3.63159 |
|  | XP\_065063258\_5622-model31.2 | -3.61484 |
|  | XP\_065063258\_5622-model31.3 | -3.4169 |
| # cluster 32 - Size: 1 |  |  |
|  | XP\_065063258\_5622-model32 | -3.83903 |
| # cluster 33 - Size: 1 |  |  |
|  | XP\_065063258\_5622-model33 | -3.80673 |
| # cluster 34 - Size: 4 |  |  |
|  | XP\_065063258\_5622-model34 | -3.80456 |
|  | XP\_065063258\_5622-model34.1 | -3.64427 |
|  | XP\_065063258\_5622-model34.2 | -3.40462 |
|  | XP\_065063258\_5622-model34.3 | -3.0521 |
| # cluster 35 - Size: 2 |  |  |
|  | XP\_065063258\_5622-model35 | -3.79203 |
|  | XP\_065063258\_5622-model35.1 | -3.33955 |
| # cluster 36 - Size: 4 |  |  |
|  | XP\_065063258\_5622-model36 | -3.78032 |
|  | XP\_065063258\_5622-model36.1 | -3.48567 |
|  | XP\_065063258\_5622-model36.2 | -3.41189 |
|  | XP\_065063258\_5622-model36.3 | -3.35813 |
| # cluster 37 - Size: 1 |  |  |
|  | XP\_065063258\_5622-model37 | -3.7403 |
| # cluster 38 - Size: 1 |  |  |
|  | XP\_065063258\_5622-model38 | -3.68321 |
| # cluster 39 - Size: 1 |  |  |
|  | XP\_065063258\_5622-model39 | -3.65562 |
| # cluster 40 - Size: 1 |  |  |
|  | XP\_065063258\_5622-model40 | -3.62768 |
| # cluster 41 - Size: 2 |  |  |
|  | XP\_065063258\_5622-model41 | -3.61604 |
|  | XP\_065063258\_5622-model41.1 | -3.35789 |
| # cluster 42 - Size: 1 |  |  |
|  | XP\_065063258\_5622-model42 | -3.60547 |
| # cluster 43 - Size: 1 |  |  |
|  | XP\_065063258\_5622-model43 | -3.58552 |
| # cluster 44 - Size: 4 |  |  |
|  | XP\_065063258\_5622-model44 | -3.54244 |
|  | XP\_065063258\_5622-model44.1 | -3.47049 |
|  | XP\_065063258\_5622-model44.2 | -3.40693 |
|  | XP\_065063258\_5622-model44.3 | -3.31165 |
| # cluster 45 - Size: 1 |  |  |
|  | XP\_065063258\_5622-model45 | -3.52858 |
| # cluster 46 - Size: 1 |  |  |
|  | XP\_065063258\_5622-model46 | -3.52143 |
| # cluster 47 - Size: 1 |  |  |
|  | XP\_065063258\_5622-model47 | -3.51209 |
| # cluster 48 - Size: 2 |  |  |
|  | XP\_065063258\_5622-model48 | -3.50142 |
|  | XP\_065063258\_5622-model48.1 | -3.24649 |
| # cluster 49 - Size: 2 |  |  |
|  | XP\_065063258\_5622-model49 | -3.47436 |
|  | XP\_065063258\_5622-model49.1 | -3.32337 |
| # cluster 50 - Size: 1 |  |  |
|  | XP\_065063258\_5622-model50 | -3.40542 |
| # cluster 51 - Size: 2 |  |  |
|  | XP\_065063258\_5622-model51 | -3.38497 |
|  | XP\_065063258\_5622-model51.1 | -3.01977 |
| # cluster 52 - Size: 1 |  |  |
|  | XP\_065063258\_5622-model52 | -3.34962 |
| # cluster 53 - Size: 1 |  |  |
|  | XP\_065063258\_5622-model53 | -3.23946 |
| # cluster 54 - Size: 1 |  |  |
|  | XP\_065063258\_5622-model54 | -3.19511 |
| # cluster 55 - Size: 1 |  |  |
|  | XP\_065063258\_5622-model55 | -3.15722 |
|  |  |  |
